# Delimiting CD34+ Stromal Cells/Telocytes Are Resident Mesenchymal Cells That Participate in Neovessel Formation in Skin Kaposi Sarcoma

**DOI:** 10.3390/ijms24043793

**Published:** 2023-02-14

**Authors:** Lucio Díaz-Flores, Ricardo Gutiérrez, Miriam González-Gómez, Maria del Pino García, Marta Palmas, Jose Luis Carrasco, Juan Francisco Madrid, Lucio Díaz-Flores

**Affiliations:** 1 Department of Basic Medical Sciences, Faculty of Medicine, University of La Laguna, 38071 Tenerife, Spain; 2 Instituto de Tecnologías Biomédicas de Canarias, University of La Laguna, 38071 Tenerife, Spain; 3 Department of Pathology, Eurofins Megalab–Hospiten Hospitals, 38100 Tenerife, Spain; 4 Department of Cell Biology and Histology, School of Medicine, Campus of International Excellence “Campus Mare Nostrum”, IMIB-Arrixaca, University of Murcia, 30100 Murcia, Spain

**Keywords:** Kaposi sarcoma, mesenchymal/stromal cells, CD34+ stromal cells/telocytes, intussusceptive angiogenesis, intussusceptive lymphangiogenesis

## Abstract

Kaposi sarcoma (KS) is an angioproliferative lesion in which two main KS cell sources are currently sustained: endothelial cells (ECs) and mesenchymal/stromal cells. Our objective is to establish the tissue location, characteristics and transdifferentiation steps to the KS cells of the latter. For this purpose, we studied specimens of 49 cases of cutaneous KS using immunochemistry and confocal and electron microscopy. The results showed that delimiting CD34+ stromal cells/Telocytes (CD34+SCs/TCs) in the external layer of the pre-existing blood vessels and around skin appendages form small convergent lumens, express markers for ECs of blood and lymphatic vessels, share ultrastructural characteristics with ECs and participate in the origin of two main types of neovessels, the evolution of which gives rise to lymphangiomatous or spindle-cell patterns—the substrate of the main KS histopathological variants. Intraluminal folds and pillars (papillae) are formed in the neovessels, which suggests they increase by vessel splitting (intussusceptive angiogenesis and intussusceptive lymphangiogenesis). In conclusion, delimiting CD34+SCs/TCs are mesenchymal/stromal cells that can transdifferentiate into KS ECs, participating in the formation of two types of neovessels. The subsequent growth of the latter involves intussusceptive mechanisms, originating several KS variants. These findings are of histogenic, clinical and therapeutic interest.

## 1. Introduction

Kaposi sarcoma (KS) is a vascular tumor of variable behavior that can affect different organs, with a higher incidence in the skin. Since it was first described by Kaposi in 1872 [1], several cell origins have been proposed for KS. It is generally considered that, in its early stages, KS is a reactive angioproliferative lesion with the possibility of evolving into true neoplasia [2,3,4,5] and that the two main cell origins are endothelial cells (ECs) and mesenchymal/stromal cells. The endothelial origin, above all lymphatic endothelium, is based on the KS cell expression for ECs of both lymphatic and blood vessel markers [6,7]. The mesenchymal/stromal cell origin is based on (a) the transdifferentiation demonstrated in other conditions of stromal cells/fibroblasts toward ECs, which can occur directly or be reprogrammed through specific transcription factors [8,9,10,11,12,13], and (b) several studies, including immunologic and genetic observations, which contribute a specific mesenchymal-to-endothelial transition in KS associated with herpesvirus infection [14,15,16,17]. KS herpesvirus-induced EC reprogramming supports viral persistence and contributes to KS tumorigenesis. However, the precise location and type of transforming mesenchymal/stromal resident (native) cells that have the ability to originate endothelial KS cells and the morphologic findings during neovessel formation and evolution require new studies. Observations for this purpose should be made in the early stages of KS lesions, in which neovessel formation, and therefore cell transdifferentiation, has begun. Indeed, in addition to the lymphedematous, lymphangiomatous, bullous, telangiectatic, ecchymotic, glomeruloid, intravascular, hyperkeratotic, keloidal, and anaplastic or pleomorphic variants, three classical and usual types of cutaneous KS related to disease progression have been described: patch or macular, plaque, and nodular [5,18,19]. Taking into account the above, a study from the patch to the plaque to the nodular stage, with initial subtle vasoformative processes, lymphangiomatous changes and progressive sarcomatous lesions, is of interest to assess the possible mesenchymal progenitors of KS cells and the subsequent events of the neoformative vessels.

Among the tissue-resident stromal cells, in order to explore their capacity as precursor mesenchymal cells in KS, are CD34+ stromal cells/Telocytes (CD34+SCs/TCs), which are located in the stroma of multiple anatomical sites [20,21]. These cells are more abundant around vessels and glands, where they have received several names, including delimiting CD34+SCs/TCs, delimiting stromal cells, delimiting fibroblasts, adventitial cells, adventitial fibroblastic cells and adventitial progenitor cells [22]. In the skin, delimiting CD34+SCs/TCs are arranged around the pericytic microvasculature (pericytes and CD34+SCs/TCs as perivascular cells), larger caliber vessels (adventitial cells) and skin appendages (delimiting cells of hair follicles, arrector pili muscle and sweat and sebaceous glands) [22,23,24,25,26,27,28,29,30,31]. CD34+SCs/TCs show a small cellular body (nuclear or somatic region) and bipolar or multipolar, thin and long moniliform cytoplasmic processes (telopodes), which communicate with those of neighboring CD34+SCs/TCs and present alternating slender segments (podomeres) and dilations (podoms) [20,21]. These processes are flat with a wing-like image in 3D reconstructions [24] or in very occasional random histologic sections [22,31]. Thus, each CD34+/SCs/TC has a lamellar or velamentous morphology (bat-like cells), which together form a labyrinthine system (perforated basket image). In addition to CD34 expression, CD34+SCs/TCs are positive for PDGFR and vimentin and negative for CD31, a differential fact in normal conditions with respect to ECs, which express CD31 [32,33]. However, loss and gain of immunomarkers can occur in CD34+SCs/TCs in some physiological and pathological processes, in vivo or during in vitro passages [22,34,35,36,37]. Among the functions hypothesized for CD34+SCs/TCs are intercellular communication, structural support and the formation of scaffolds, as well as the control of other cell types and organization of the extracellular matrix, immunomodulation, endocytosis, guidance to migration, stem cell modulation, progenitor capacity and participation in regeneration, repair and tumor stroma formation [20,21,23,31,38,39,40,41,42,43,44,45,46,47,48,49,50,51,52].

Given the above, the first objective of this study on KS skin lesions is to assess whether delimiting CD34+SCs/TCs, located in the outer layer of pre-existing blood vessels and around skin appendages, are mesenchymal/stromal cells with a capacity for transdifferentiation into neovessel ECs that express markers for both blood and lymphatic vessels. A better knowledge of the type and location of resident stromal precursor cells with vasoformative capacity is of histogenic, therapeutic and experimental interest, especially for assessing their modulation during tumor development and for obtaining them for in vitro studies. Subsequently, we will follow the events of the neovessels in the evolutionary stages of KS. This information is also of clinical interest since it could be the basis for future studies in larger series of cutaneous KS cases that confirm a possible evolution toward different variants and the prognostic projection of early lesions. For these purposes, in cases of KS (*n*: 49), using conventional techniques and procedures for observation by immunochemistry, immunofluorescence and electron microscopy, we will take into account: (a) the general characteristics of the main evolutionary stages of KS; (b) the location and characteristics of the CD34+SCs/TCs in the outer layer of pre-existing blood vessels and around skin appendages, with or without perivascular inflammatory infiltrate, and with or without formation of neovessels; and (c) the findings supporting the possible role of delimiting CD34+SCs/TCs on the formation of neovessels and their behavior in the evolutionary stages of KS.

## 2. Results

### 2.1. General Characteristics of KS in Patch, Plaque and Nodular Stages

**Figure 1:**
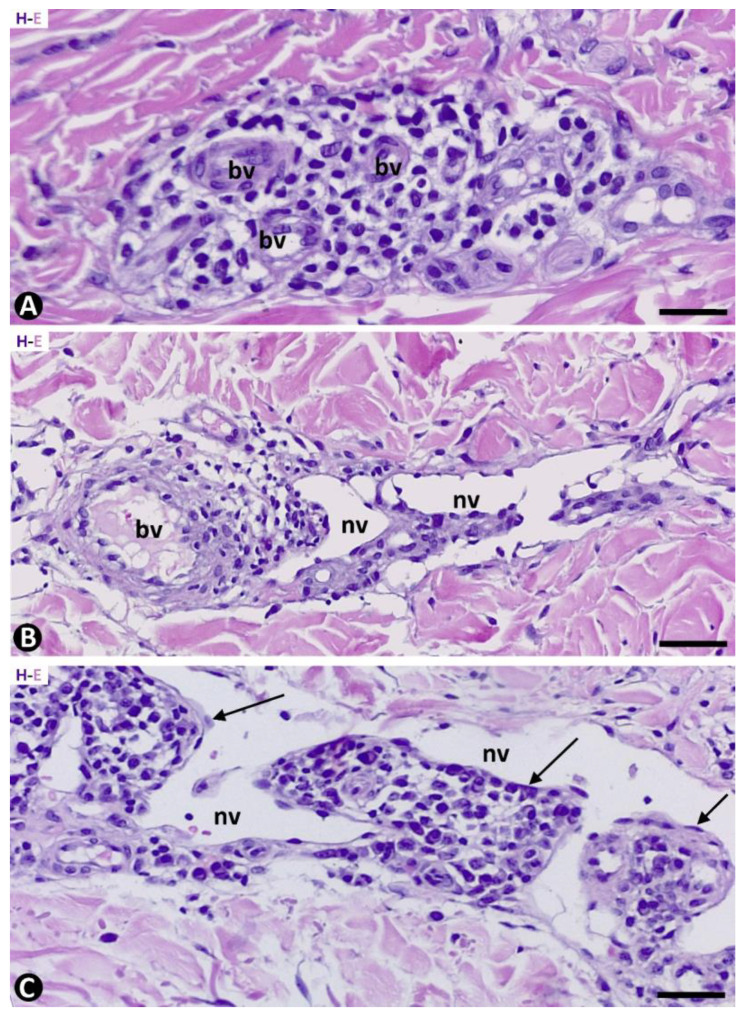
Inflammatory infiltrate (**A**) and associated newly formed vessels ((**B**,**C**), nv) around blood vessels ((**A**–**C**), bv) are observed in the patch stage of KS. Note in B and C the protrusion of the blood vessels and the perivascular inflammatory infiltrate in the lumen of the neovessels, giving rise to the promontory sign ((**C**), arrows). Hematoxylin and eosin. Bar: (**A**): 30 µm, (**B**): 50 µm, (**C**): 40 µm.

In the patch stage, edema, mild to moderate inflammatory infiltrate, and newly formed vessels could be observed by conventional techniques with hematoxylin–eosin staining (Figure 1). The perivascular inflammatory infiltrate (Figure 1A) was composed of mononuclear cells, predominantly lymphocytes and plasma cells, and varying numbers of macrophages. The newly formed vessels were mainly located around the pre-existing blood vessels, with or without perivascular inflammatory infiltrate (Figure 1B). Newly formed vessels were also present around skin appendages and occasionally in the intervening dermis. Protrusion of pre-existing blood vessels, with or without perivascular inflammatory infiltrate, into the lumen of neovessels gave rise to the promontory sign (Figure 1C). Red blood cell extravasation occurs with variable intensity, and siderophages can be seen.

**Figure 2:**
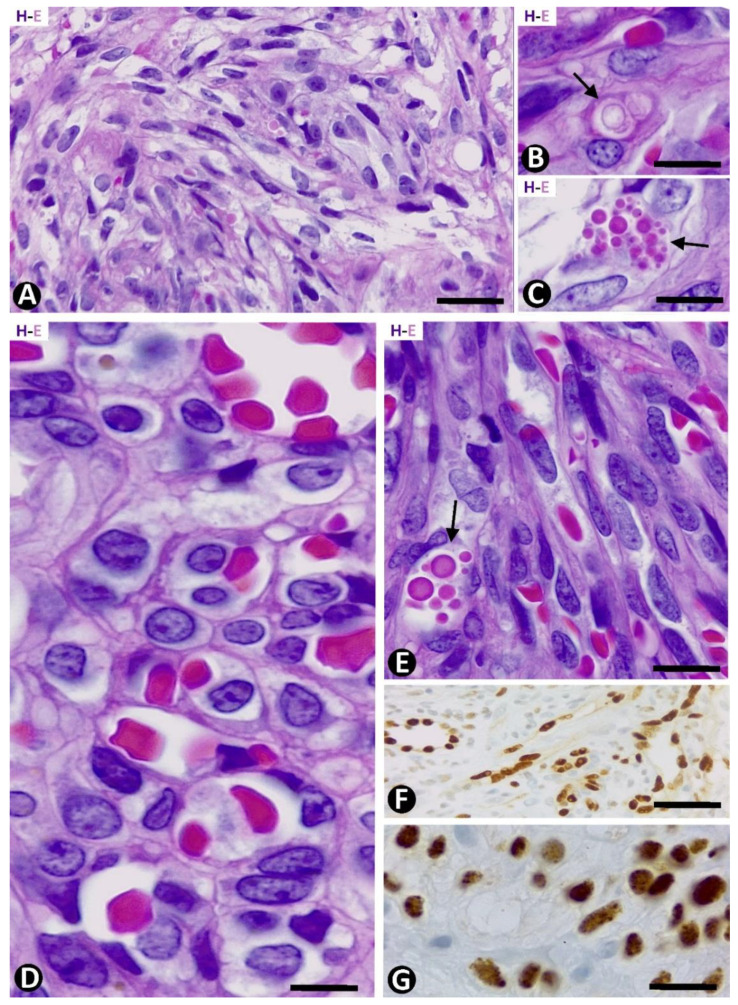
(**A**–**C**): KS in the plaque stage. Fusiform cells forming narrow cords with virtual or erythrocyte-occupied lumens are observed in (**A**). Note degraded red blood cells incorporated in the cytoplasm of an EC in (**B**) (arrow) and hyaline bodies in (**C**) (arrow). (**D**,**E**): KS in the nodular stage. High cellular fascicles of spindle cells (**E**) with intraluminal red blood cells. Note a honeycomb-like pattern in a cross-section in (**D**) and hyaline bodies in (**E**) (arrow). (**F**): Nuclear expression for ERG in KS ECs. (**G**): Human herpesvirus 8 (HHV-8) expression. (**A**–**E**) Hematoxylin and eosin. (**F**,**G**): Immunochemistry for ERG and HHV-8, respectively. Bar: (**A**,**F**): 20 µm; (**B**,**C**,**E**,**G**): 15 µm; (**D**): 10 µm.

The plaque stage revealed an increase in the events of the patch stage, with greater cellularity and an inflammatory infiltrate presenting plasma cells, lymphocytes, and varying numbers of siderophages. The vascular component was more extensive and diffuse than in the patch stage and was also made up of pre-existing and newly formed vessels. The latter appeared as dissecting vascular spaces, with or without the promontory sign, or as fascicles formed by highly cellular narrow cords showing virtual, empty or erythrocyte-occupied lumen (Figure 2A). The cells in the narrow cords showed a spindle-shaped morphology with sparse mitotic figures and without nuclear pleomorphism. Red blood cell extravasation with the presence of siderophages, intracytoplasmic incorporation of red blood cells by ECs (autolumination phenomenon), and intra or extracellular hyaline globules, probably corresponding to modified red blood cells, was observed with relative frequency (Figure 2B,C).

The nodular stage revealed KS spindle cells, which replaced the dermal collagen and presented increased mitotic activity (6–18 mitoses/10 high-power fields) in the anaplastic KS. In the cross-sections, the KS cells appear around small or varying caliber lumens with the intraluminal presence of red blood cells; the lesion adopts a honeycomb-like pattern (Figure 2D). When longitudinally sectioned, the KS spindle cells showed an elongated nucleus and delimited lumens, virtual or with rows of red blood cells (Figure 2E). The intracytoplasmic degraded red blood cells and hyaline bodies were present in some KS cells (Figure 2E).

Nuclear expression for the endothelial marker ERG (Figure 2F) and for the Herpes virus human 8 (HHV-8) (Figure 2G) was observed in all cases.

Next, we will pay particular attention to the pre-existing blood vessels and complex structures (associated vessels and skin appendages) in KS lesions and to the newly formed vessels to establish the location, characteristics, relationship and participation of CD34+SCs/TCs in these neovessels.

### 2.2. Pre-Existing (Native) Blood Vessels and Their Relationship with the Inflammatory Infiltrate and Newly Formed Vessels in the Skin Affected by KS in Patch and Plaque Stages

A close relationship between the pre-existing blood vessels, inflammatory infiltrate, and newly formed vessels was observed in KS of the skin. The pre-existing blood vessels, with or without perivascular inflammatory infiltrate, did or did not present perivascular neovessels. Therefore, the pre-existing blood vessels in early cutaneous KS lesions were (a) apparently non-affected (without perivascular inflammatory infiltrate or perivascular neovessels), (b) with perivascular inflammatory infiltrate, and (c) with perivascular neovessels, presenting perivascular inflammatory infiltrate or not. Pre-existing vessels with perivascular inflammatory infiltrate or perivascular neovessels (with or without inflammatory infiltrate) were in similar proportions and predominated over apparently unaffected pre-existing blood vessels.

#### 2.2.1. CD34+SCs/TCs in Pre-Existing Blood Vessels without Perivascular Inflammatory Infiltrate or New Vessels (Apparently Non-Affected Blood Vessels)

**Figure 3:**
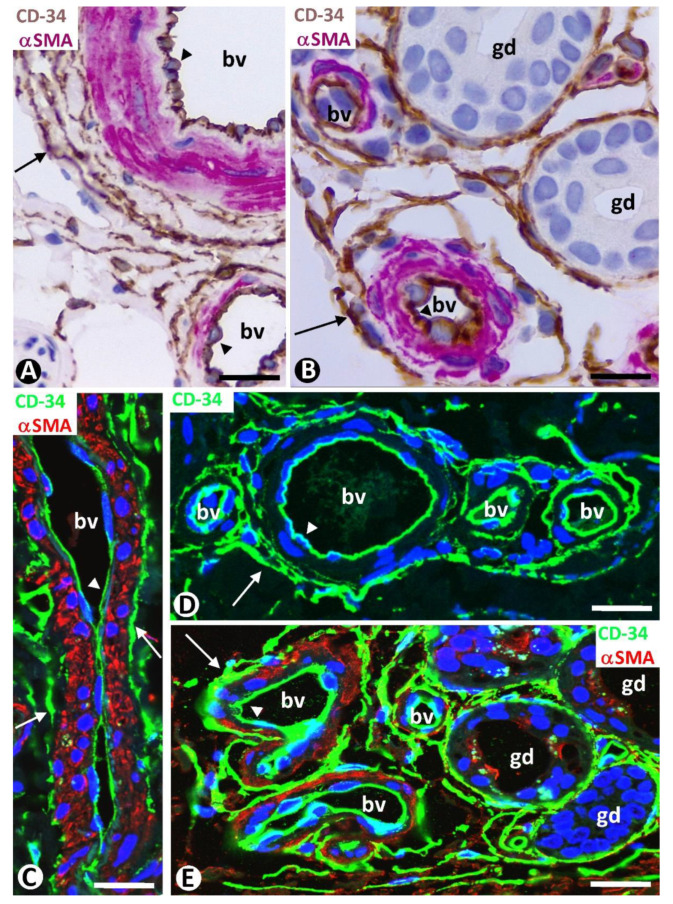
Pre-existing blood vessels (bv in (**A**,**C**,**D**)) and complex structures with vessels and glands (bv and gd, respectively, in (**B**,**E**)) in areas without perivascular newly formed vessels or inflammatory infiltrate. Three layers in the pre-existing blood vessels are observed in (**A**,**B**), with double immunochemistry for CD34 and αSMA. Intima layer, formed by CD34+ ECs (arrowhead) (brown); media layer, made up of αSMA+ pericytes or vascular smooth muscle cells (red); and external layer with CD34+SCs/TCs (arrows) (brown). Similar images in immunofluorescence in (**C**,**E**): Intima layer (arrowhead) (green), media layer (red), and external layer with CD34+SCs/TCs (arrows) (green). In (**D**), CD34 expression is highlighted in both ECs (arrowhead) and CD34+SCs (arrows) by immunofluorescence, with only CD34 (green). Note the formation of a labyrinthine system by CD34+SCs/TCs around complex structures (blood vessels and glands) in (**B**,**E**). Counterstain is with hematoxylin in (**A**,**B**) and with DAPI in (**C**–**E**). Bar: (**A**,**B**): 20 µm; (**C**,**D**): 35 µm; (**E**): 25 µm.

Double staining for CD34 and αsmooth muscle actin (αSMA) revealed three layers formed by the CD34+ ECs (intima layer), αSMA+ pericytes or vascular smooth muscle cells (media layer), and delimiting CD34+SCs/TCs (external layer) in apparently non-affected pre-existing blood vessels of the two horizontal vascular plexuses and those connecting them in the skin (Figure 3). Depending on vessel caliber, one or several sheets of delimiting CD34+SCs/TCs were observed (Figure 3A–D). An exception was the small vessels located in the most superficial areas of the dermal papillae, in which CD34+SCs/TCs were absent. A labyrinthine system of CD34+SCs/TCs was seen between and around the complex structures formed by skin appendages and blood vessels (Figure 3B,E). In addition to this delimiting arrangement, CD34+SCs/TCs could extend some of their processes into surrounding tissues.

#### 2.2.2. CD34+SCs/TCs in Pre-Existing Blood Vessels with Perivascular Inflammatory Infiltrate

**Figure 4:**
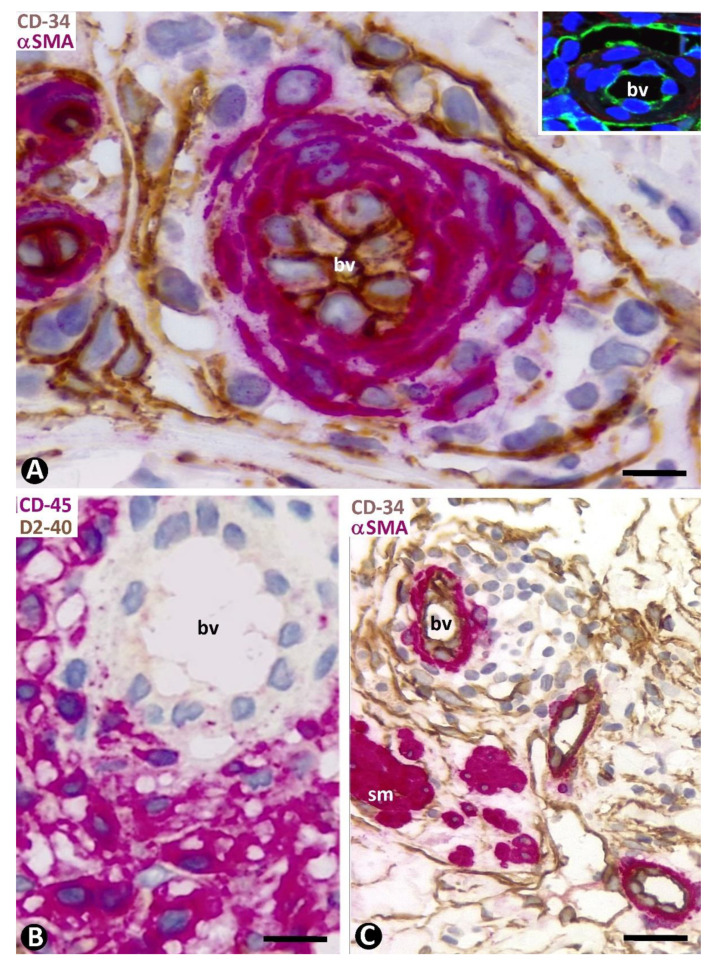
Pre-existing blood vessels with perivascular inflammatory infiltrate. (**A**): Inflammatory cells are observed between CD34+SCs/TCs (brown) and mural cells (red), respectively, located in the external and media layer of a pre-existing blood vessel (bv). In the insert, a similar image is in immunofluorescence for CD34. (**B**): Expression of CD45 by the inflammatory cells (red). (**C**): Perivascular inflammatory infiltrate like that of A, in a complex structure (presence of smooth muscle red, sm). (**A**,**C**): Double immunochemistry for CD34 (brown) and αSMA (red). Hematoxylin counterstain. Insert in (**A**): Immunofluorescence for CD34. DAPI counterstain. (**B**): Double immunochemistry for CD45 (red) and D2-40 (not expressed). Hematoxylin counterstain. Bar: (**A**): 10 µm; (**B**): 20 µm; (**C**): 35 µm.

The perivascular inflammatory infiltrate in the pre-existing blood vessels was observed in the space between the CD34+SCs/TCs and pericytes or vascular smooth muscle cells (Figure 4A). When CD34+SCs/TCs were arranged in several sheets, the inflammatory infiltrate was located between and delimited by them. The inflammatory cells showed expression for CD45 (Figure 4B), and the inflammatory infiltrate predominated around the vessels in the complex structures (Figure 4C).

#### 2.2.3. CD34+SCs/TCs in Pre-Existing Blood Vessels with Perivascular Neovessels

**Figure 5:**
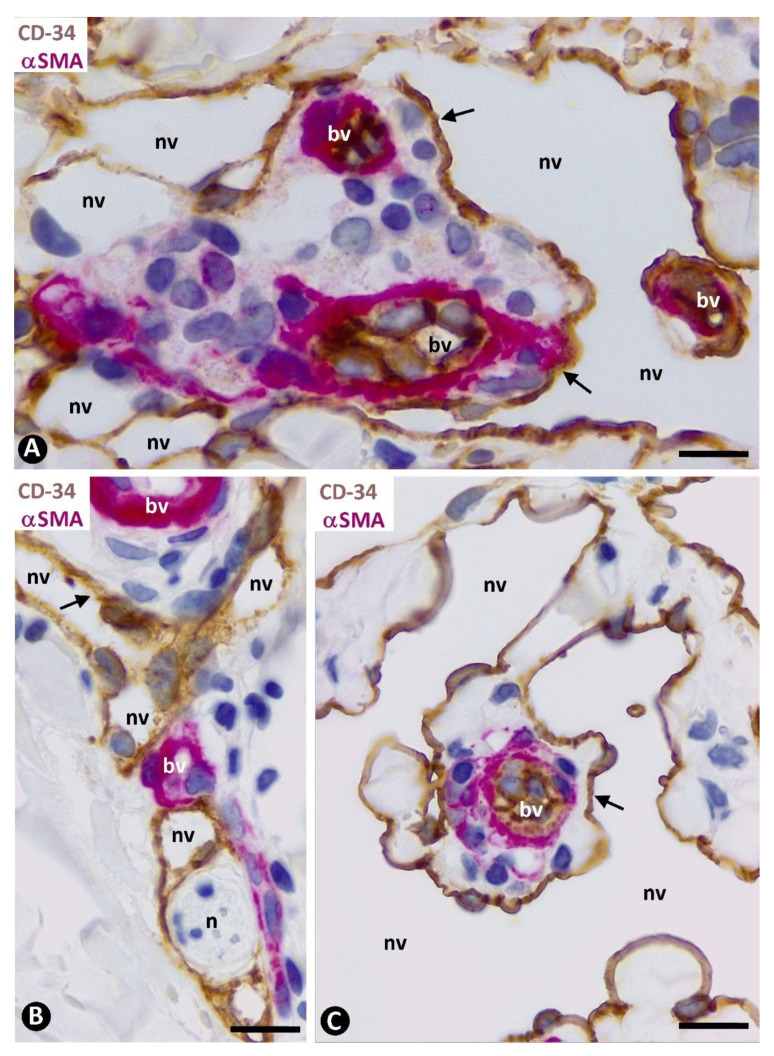
(**A**–**C**). Neovessels (nv, corresponding to type 1) around pre-existing blood vessels (bv). The precise location of the neovessel ECs coincides with that of the CD34+SCs/TCs in the apparently non-affected blood vessels or with perivascular inflammatory infiltrate (arrows) (compare with Figure 3 and Figure 4). Note that the pre-existing blood vessels (bv, with αSMA+ pericytes or vascular smooth muscle cells, red) are totally or partially surrounded by the neovessels (without pericytes or vascular smooth muscle cells), giving rise to the image of a vessel within a vessel (promontory sign). Double immunochemistry for CD34 and αSMA. Hematoxylin counterstain. Bar: (**A**): 15 µm; (**B**,**C**): 20 µm.

Neovessels were mainly observed around the pre-existing blood vessels (Figure 5), predominantly in the dermal, superficial horizontal vascular plexus. A differential fact between the pre-existing blood vessels and neovessels was the presence of αSMA+ pericytes or vascular smooth muscle cells in the former and their absence in the neovessels (Figure 5).

The precise location of the neovessels coincided with that of the previously delimiting CD34+/SCs/TCs around the blood vessels (Figure 5), suggesting a change from the CD34+SCs/TCs to neovessel ECs. Likewise, intraluminal folds and pillars were observed in the neovessels. Given this, we will first consider the main types and characteristics of the initial neovessels, and of the intraluminal folds and pillars, paying particular attention to the findings supporting their formation from delimiting CD34+SCs/TCs. Next, we will briefly consider the evolution of the neovessels in the advanced stages of KS.

### 2.3. Initial Neovessels in Early KS Lesions

**Figure 6:**
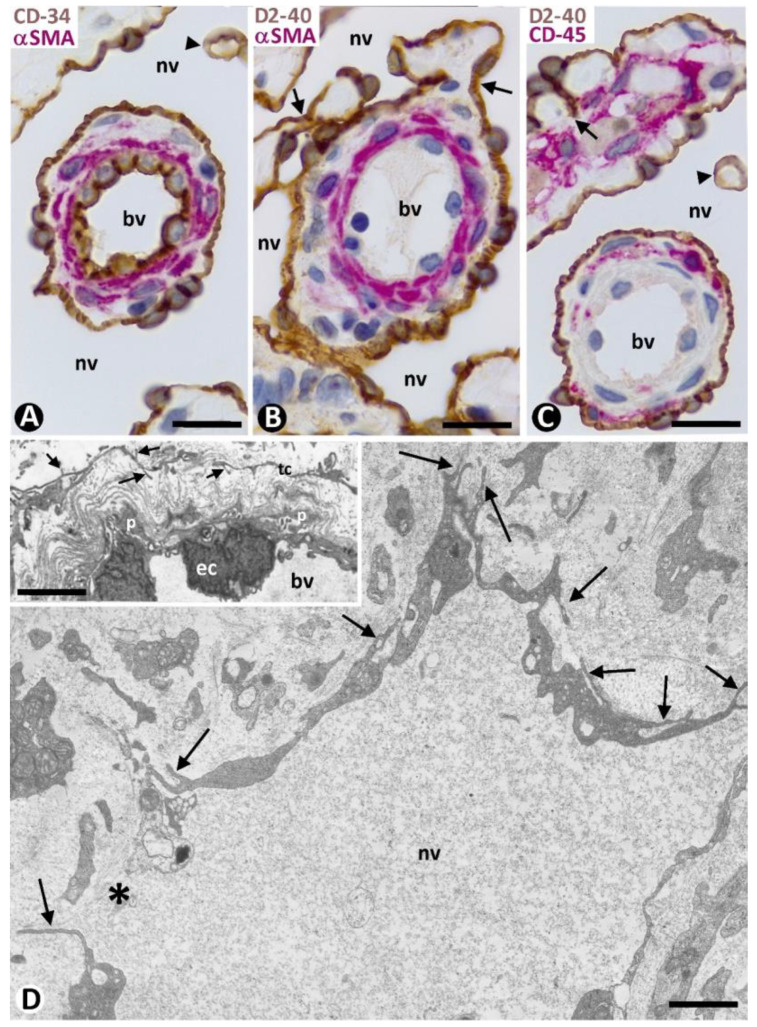
Characteristics of the type 1 neovessel (nv) around pre-existing blood vessels (bv). (**A**–**C**): Observe that ECs of neovessels express CD34 ((**A**), brown) (a marker of blood vessels ECs) and D2-40 ((**B**,**C**), brown) (a marker of lymphatic ECs), while ECs of pre-existing blood vessels only express CD34 ((**A**), brown). In (**A**,**B**), the pericytes and vascular smooth muscle cells express αSMA (red). In (**C**), CD45+ inflammatory cells (red) are seen in the interstitium and around a pre-existing blood vessel. (**D**): Ultrastructural characteristics of a type 1 neovessel. Alternating thick and thin regions, discontinuity zones (asterisk), and processes extending to the interstitium (arrows) are observed in ECs, resembling telocyte characteristics. In the insert, ultrastructural characteristics of delimiting telocytes (arrows) in a pre-existing blood vessel (bv) with intimal ECs (ec) and pericytes (p). (**A**): Double immunochemistry for CD34 (brown) and αSMA (red). (**B**): Double immunochemistry for D2-40 (brown) and αSMA (red). (**C**): Double immunochemistry for D2-40 (brown) and CD45 (red). (**A**–**C**): Hematoxylin counterstain. (**D**) and insert: Ultrathin sections. Uranyl acetate and lead citrate. Bar: (**A**–**C**): 20 µm; (**D**): 2.5 µm.

Although several patterns of neovessels were present in the patch and plaque stages of KS, two main types were seen (types 1 and 2) (Table 1). Both types of neovessels were predominantly arranged around pre-existing blood vessels, coinciding in a location with that previously held by CD34+SCs/TCs (Figure 5 and Figure 6A–C), as mentioned above. Next, we consider the characteristics of type 1 and 2 neovessels.

#### 2.3.1. Characteristics of Type 1 Neovessels

**Figure 7:**
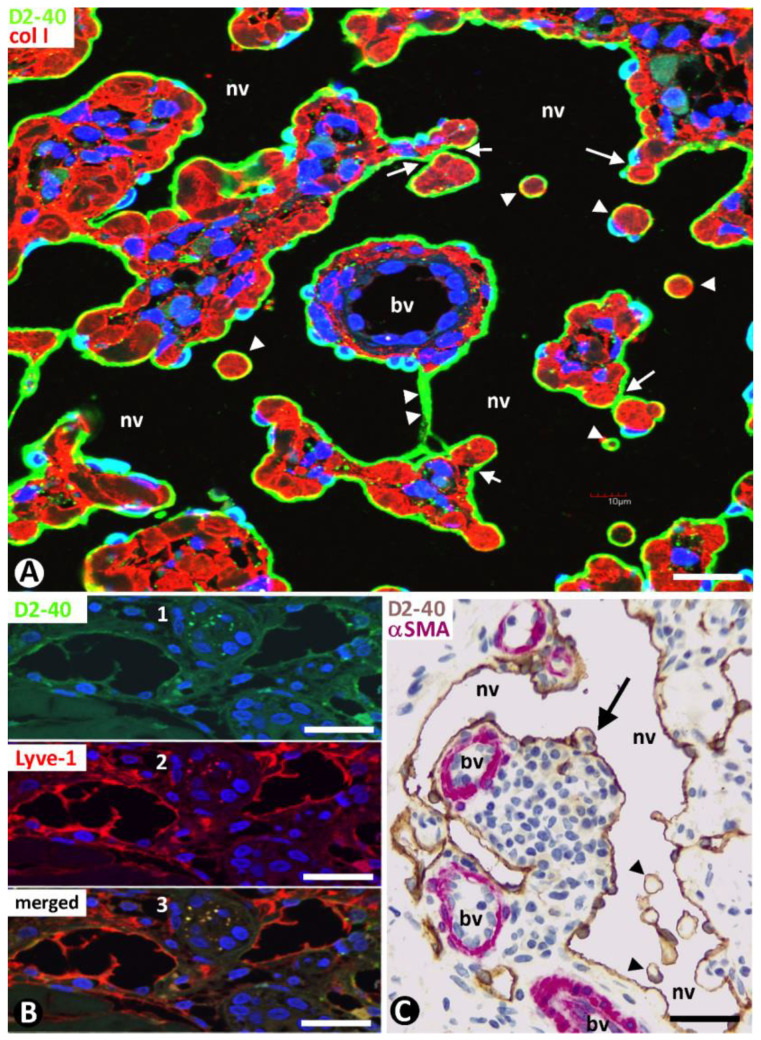
(**A**): Numerous pillars (arrowheads) and folds (thicker than pillars) in the wide lumen of a neovessel type 1 (nv) are observed by double immunofluorescence for D2-40 (green) and collagen I (red). Note that each pillar and fold shows a cover and a core. The covers are formed by D2-40+ ECs, and the cores present collagen I. An endothelial bridge or nascent pillar (double arrowhead) is observed between two folds. (**B**): D2-40 (1, green) and Lyve-1 (2, red) co-expression (3, merged) in neovessels. (**C**): Blood vessels (bv) in a fold with inflammatory cells and various pillars (arrowheads). (**A**): Double immunofluorescence for D2- 40 (green) and collagen I (red). DAPI counterstain. (**B**): Immunofluorescence for D2-40 (1, green), Lyve-1 (2, red), and merged (3). (**C**): Double immunochemistry for D2-40 (brown) and αSMA (red). Hematoxylin counterstain. Bar: (**A**): 25 µm; (**B**): 35 µm; (**C**): 50 µm.

Type 1 neovessels showed lumens of differing sizes and configurations, ranging from slit-like vascular spaces to wide sacs with a lymphangiomatous appearance (Figure 5). Generally, the different neovessel lumens converged, giving rise to paths that varied in aspect. The lumens were lined by a monolayer of flattened ECs, which showed expression for markers of both blood and lymphatic vessels (Figure 5 and Figure 6). Thus, in addition to positivity for CD34 (a marker for blood vessel ECs) (Figure 5 and Figure 6A), the ECs of the neovessels also expressed D2-40 (a marker for lymphatic vessel ECs) (Figure 6B,C), while the ECs of the pre-existing vessels were positive for CD34 (Figure 5 and Figure 6A) and negative for D2-40 (Figure 6B,C). The difference in marker expression in the ECs, according to blood or lymphatic vessels, was evident when each marker was combined with αSMA expression, as indicated above. Likewise, when present, the CD45+ perivascular inflammatory cells were distinguished by double immunostaining (for D2-40 and CD45) (Figure 6C). Ultrastructurally, neovessel ECs showed alternating thick and thin regions, resembling the podomeres and podoms of telocytes, respectively (Figure 6D and compare this with the insert). Discontinuity zones, small intercellular junctions and cell processes extending to the interstitium were also observed in neovessel ECs (Figure 6D). Pillars and folds, with a cover and a core, were present in the lumen of neovessels. The cover of pillars and folds was formed by ECs with the same characteristics as those lining the neovessels, and the core contained packed collagen fibers, which were positive for collagen I (Figure 7A). Nascent pillars formed by ECs were also seen (Figure 7A). The co-expression of D2-40 y Lyve-1 was observed in some neovessels (Figure 7B), and CD31 expression was seen in the ECs of both pre-existing blood vessels and neovessels. The core of larger intraluminal folds could present blood vessels with or without perivascular inflammatory infiltrate (blood vessels within neovessels, the promontory sign) (Figure 7C).

#### 2.3.2. Characteristics of Type 2 Neovessels

**Figure 8:**
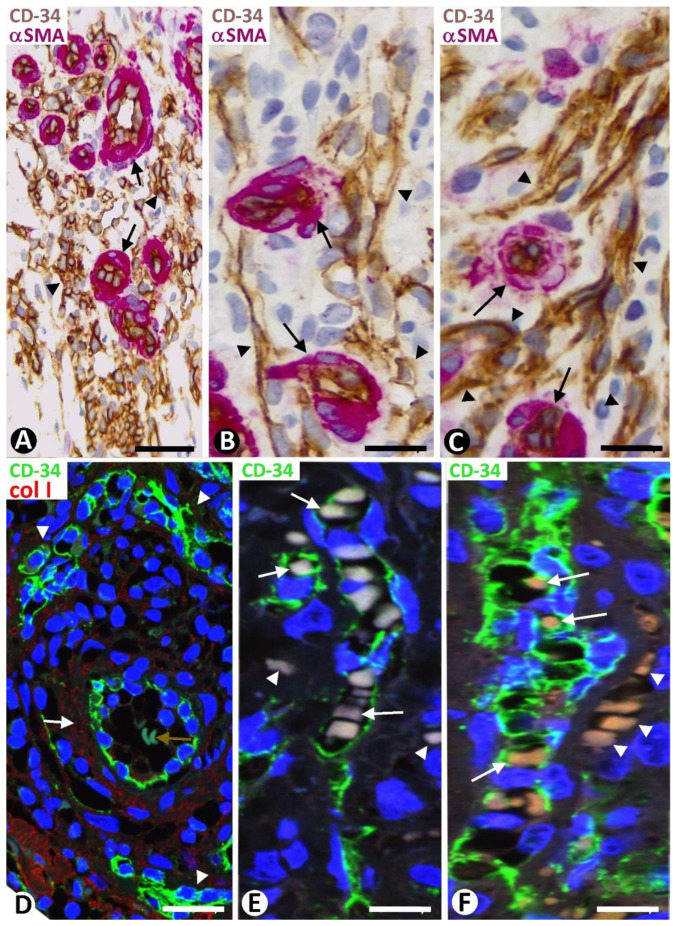
Type 2 neovessels in plaque stage of KS. (**A**–**D**): CD34+ neovessel ECs (brown in (**A**–**C**) and green in (**D**)) (arrowheads) are observed around pre-existing blood vessels in which αSMA+ pericytes and vascular smooth muscle cells are seen (red, arrows). Note that neovessel ECs are present where delimiting CD34+SCs/TCs were located in non-affected vessels. (**E**,**F**): Red blood cells are observed in the neovessel lumen (arrows) and the interstitium (arrowheads). (**A**–**C**): Double immunochemistry for CD34 (brown) and αSMA (red). Hematoxylin counterstain. (**D**): Double immunofluorescence for CD34 (green) and collagen I (red). (**E**,**F**): Immunofluorescence for CD34. DAPI counterstain. Bar: (**A**,**D**): 35 µm; (**B**,**C**,**E**,**F**): 20 µm.

**Figure 9:**
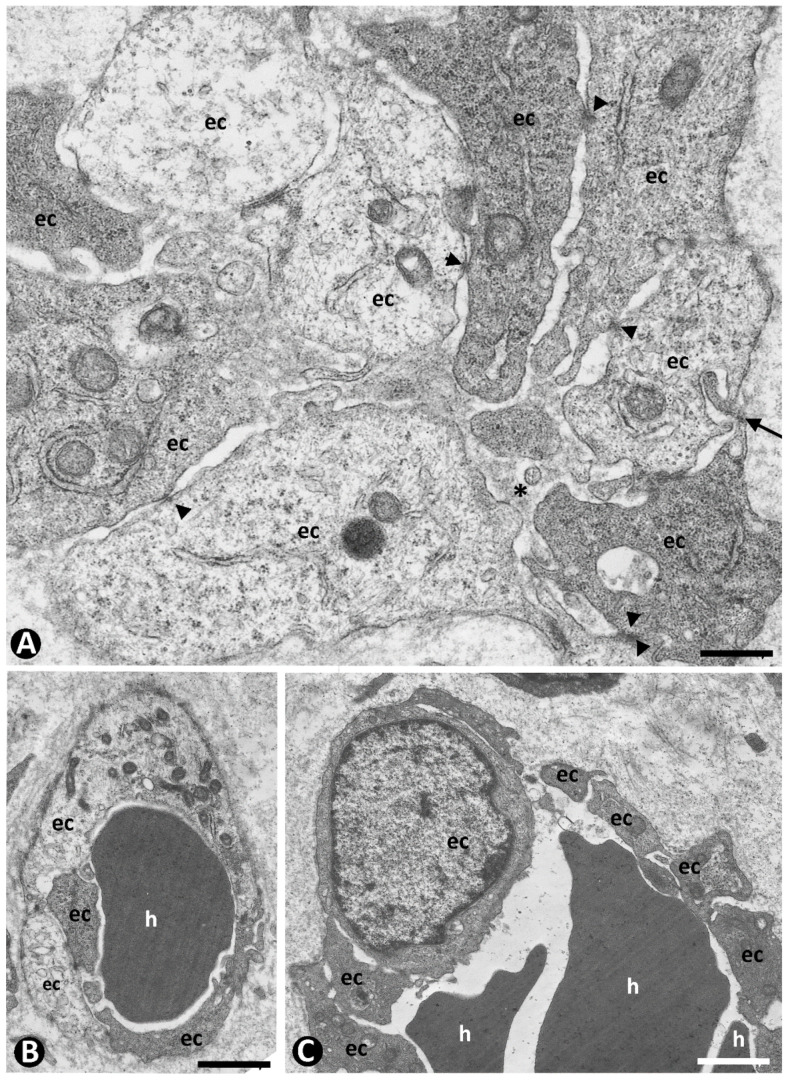
Ultrastructural characteristics of type 2 neovessels. ECs with discontinuities, different electron densities, small interendothelial junctions (arrowheads), and a peg-and-socket junction ((**A**), arrow) form neovessels with virtual lumen ((**A**), asterisk) and one (**B**) or several. (**C**) Red blood cells (h). Ultrathin sections. Uranyl acetate and lead citrate. Bar: (**A**): 0.7 µm; (**B**): 1.5 µm; (**C**): 1 µm.

Type 2 neovessels were more numerous in the plaque stage and appeared located around blood vessels in the previous location of the CD34+SCs/TCs, as occurred in type 1 vessels (Figure 8A–D). Most type 2 neovessels presented virtual or small lumens and a fascicular appearance when sectioned longitudinally (Figure 8B–D). Depending on the orientation and plane of sectioning, their ECs had a spindle or oval morphology and were generally more voluminous than those of type 1 vessels. Type 2 neovessel ECs also expressed markers for ECs of both blood and lymphatic vessels. With varying frequencies, one or several red blood cells were observed in the small lumen of these neovessels (Figure 8E,F). The red blood cells appeared, forming rows when the vessels were sectioned longitudinally (Figure 8E). Neovessels with more dilated lumens and presenting with numerous intra- and extraluminal red blood cells and the occasional pillars were intermingled with those with smaller or virtual lumens and were occasionally numerous. Ultrastructurally, some of these neovessels showed virtual lumens, which were not demonstrated by other procedures (Figure 9A). The ECs of type 2 neovessels with discontinuities, different electron densities and small interendothelial adherent and peg-and-socket junctions were also frequently observed under the electron microscope (Figure 9A–C).

#### 2.3.3. Findings Supporting CD34+SC/TC Participation in the Origin of Neovessel ECs

**Figure 10:**
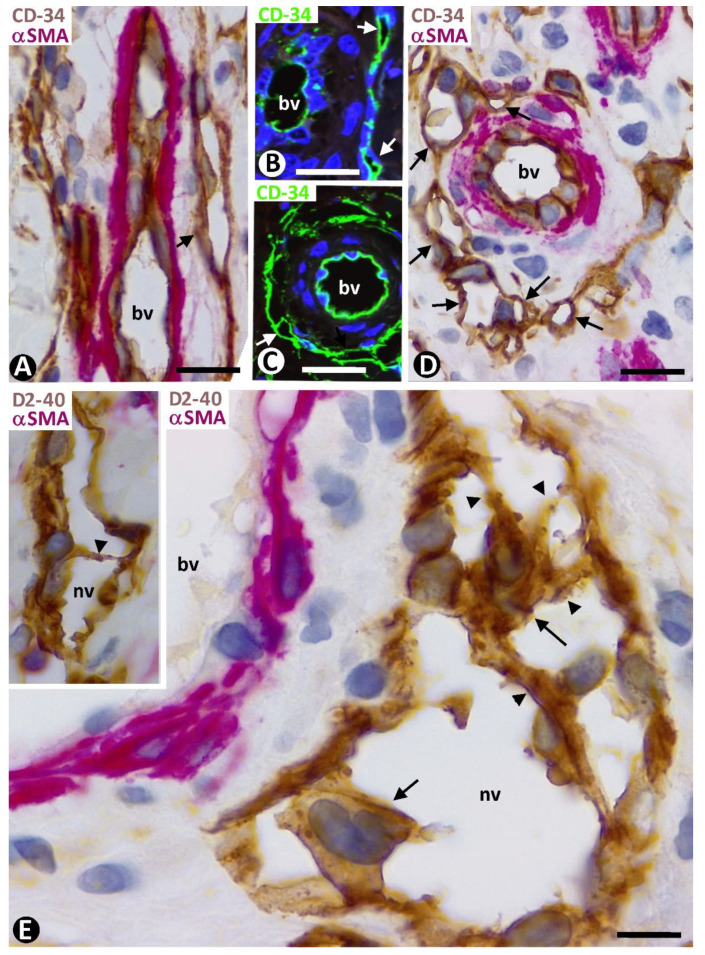
(**A**–**D**): Small neovessel lumens (arrows) lined by CD34+SCs/TCs (brown in (**A**,**D**) and green in (**B**,**C**)) located around blood vessels (bv). Note in (**D**) a greater number of small neovessel lumens. (**E**): A neovessel (nv), formed by CD34+SCs/TCs (brown), with two of them in its lumen (arrows), is observed around a blood vessel (bv) with αSMA+ mural cells (red). Note that the intraluminal CD34+SCs/TCs divide the neovessel lumen into several spaces and that one has various processes (arrowheads), retaining its stellate morphology. In the insert, another newly formed vessel with an intraluminal process of a CD34+SC/TC (arrowhead). (**A**,**D**): Double immunochemistry for CD34 and αSMA. Hematoxylin counterstain. (**B**,**C**): Immunofluorescence for CD34. DAPI counterstain. (**E**): Double immunochemistry for D2-40 and αSMA. DAPI counterstain. Bar: (**A**,**D**): 20 µm; (**B**,**C**): 35 µm; (**E**): 10 µm.

In addition to the coincidence of the neovessel EC location and that of delimiting CD34+SCs/TCs, the following findings were observed during neovessel formation. The somatic region and processes of one or more delimiting CD34+SCs/TCs folded or converged, forming occasional, isolated small vessel lumens in the early stages of KS (Figure 10A–C). These small neovessel lumens alternated with other perivascular CD34+SCs/TCs, without forming lumens. The number of neoformed vessel lumens from the CD34+SCs/TCs appeared to increase around other pre-existing blood vessels (Figure 10D). These newly formed vessel lumens could be subdivided by intraluminal CD34+SCs/TCs endowed with two or several cytoplasmic processes, which extended to different points of the neovessel wall (Figure 10E). The spaces formed in the lumen of the neovessels could also be separated by voluminous CD34+ ECs or by their processes (Figure 10E and insert).

**Figure 11:**
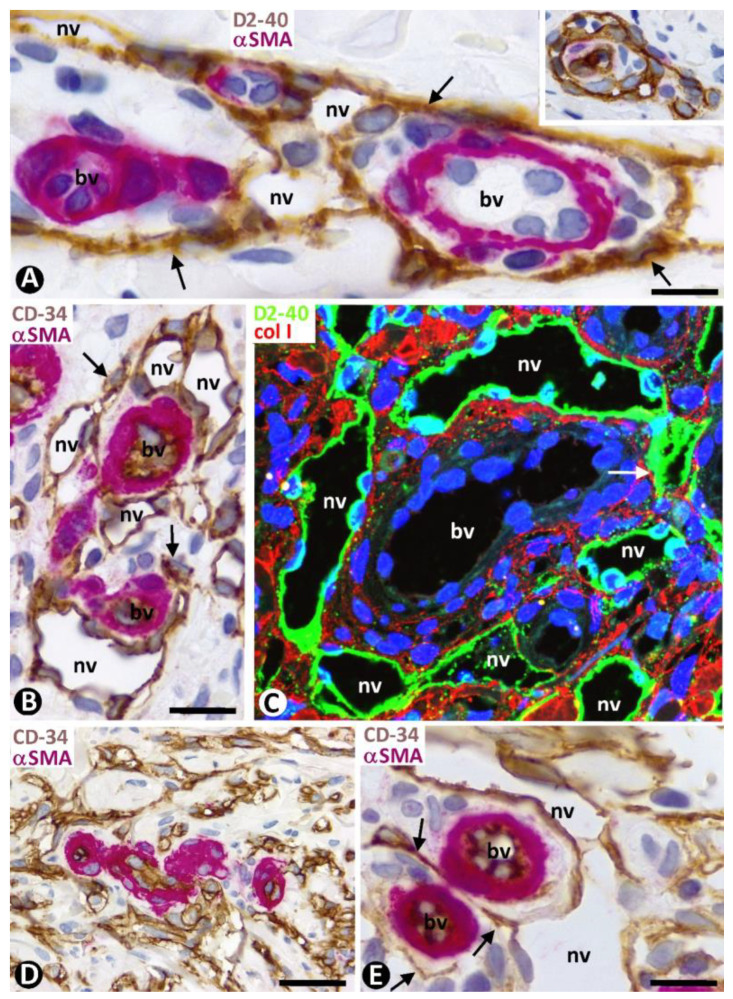
(**A**): All delimiting cells (brown, arrows) around blood vessels (bv), forming occasional and small neovessels lumen (nv), or not, are positive for D2-40. Insert of (**A**): ECs of neovessels with small lumens and interposed delimiting cells expressing CD34 (brown). (**B**,**C**): Delimiting cells (arrows) and ECs of dilated neovessel lumens (nv) expressing CD34: ((**B**), brown) and D2-40 ((**C**), green). (**D**): ECs of neovessels forming narrow cords (type 2 neovessels) (brown) around pre-existing blood vessels with pericytes (red) tangentially sectioned. (**E**): ECs of a dilated neovessel (nv, brown) continuous with a delimiting cell (brown, arrows) arranged between two pre-existing blood vessels (bv). Note that ECs of pre-existing blood vessels (bv) express CD34 ((**B**,**D**,**E**) and insert of (**A**)) but do not express D2-40 (**A**,**C**), while neovessel ECs express both markers of blood and lymphatic ECs (CD34 and D2-40). (**A**): Double immunochemistry for D2-40 and αSMA. (**B**,**D**,**E**) and insert in (**A**): Double immunochemistry for CD34 and αSMA. (**A**,**B**,**D**,**E**) and insert of (**A**): Hematoxylin counterstain. (**C**): Double immunofluorescence for D2-40 and collagen I. DAPI counterstain. Bar: (**A**,**C**): 15 µm; (**B**,**E**): 20 µm; (**D**): 35 µm.

The ECs of the initially evolved neovessels gave rise to enlarged and confluent lumens (neovessel type 1) or formed narrow cords (neovessel type 2) and showed expression for markers of both blood and lymphatic ECs. A similar expression for both types of markers was observed in the CD34+SCs/TCs interposed between the neovessels and without forming a lumen (Figure 11A–C). Although these neovessels could appear separated from the pre-existing blood vessels, well-oriented histological sections showed their relationship with the latter (Figure 11D). Likewise, the ECs of neovessels were observed in continuity with delimiting CD34+SCs/TCs arranged between pre-existing blood vessels (Figure 11E). As mentioned, the ECs of the pre-existing blood vessels did not show expression for lymphatic vessel markers, unlike the neovessels (Figure 11A,C).

#### 2.3.4. CD34+SCs/TCs during the Formation of Pillars in the Lumen of Neovessels

**Figure 12:**
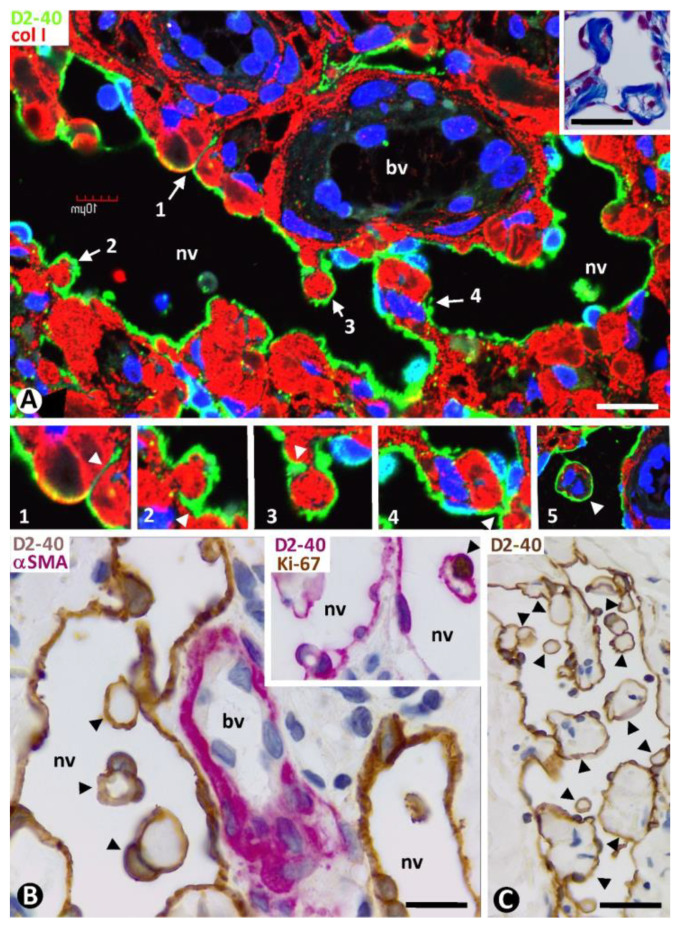
(**A**): Transformed CD34+SCs/TCs are observed lining a divided neovessel. Note processes of CD34+SCs/TCs lining neovessel cells extending between and around collagen fibers (at higher magnification in 1–4). One bridges the two points of the neovessel wall (4). (**A**) Similar structure is seen in the neovessel lumen with a pillar-like aspect (cover formed by ECs and core by collagen) (5). (**B**,**C**): Neovessels showing numerous intraluminal pillars. Insert of (**B**). Nuclear Ki-67 expression in a covering cell of a pillar. Observe Ki-67 expression in a nucleus of a pillar-covering cell (insert of (**B**)). (**A**) and 1–5: Double immunofluorescence for D2-40 and collagen I. DAPI counterstain. (**B**,**C**): Double immunochemistry for D2-40 and αSMA. Hematoxylin counterstain. Insert of (**B**): Double immunochemistry for D2-40 and Ki-67. Hematoxylin counterstain. Bar: (**A**,**B**): 10 µm; (**C**): 25 µm.

Some of the transformed CD34+SCs/TCs lining the neovessels were observed as preserving one or two of their processes that extended into the surrounding tissues (Figure 12A1–3). These processes were seen between and around collagen fibers, which they partially or totally surrounded. The surrounding processes, together with the enveloping collagen, could also appear contacting two points of the neovessel wall (Figure 12A4) or being incorporated into the neovessel lumen, acquiring a pillar aspect (Figure 12A5). These pillars were numerous in some neovessels (Figure 12B,C), and their covering cells could present Ki-67 nuclear expression (insert Figure 12B).

### 2.4. Evolution of the Neovessels in KS

Dilated type 1 neovessels, generally with numerous intraluminal folds and pillars (papillae), were observed in the lymphangiectatic and lymphangiomatous variants of KS (an increase of the already described findings for type 1 vessels). Frequently, the folds in the neovessels contained pre-existing blood vessels (a vessel inside a vessel or a promontory sign). The lumen of these type 1 neovessels appeared empty or with some inflammatory cells and/or occasional red blood cells.

**Figure 13:**
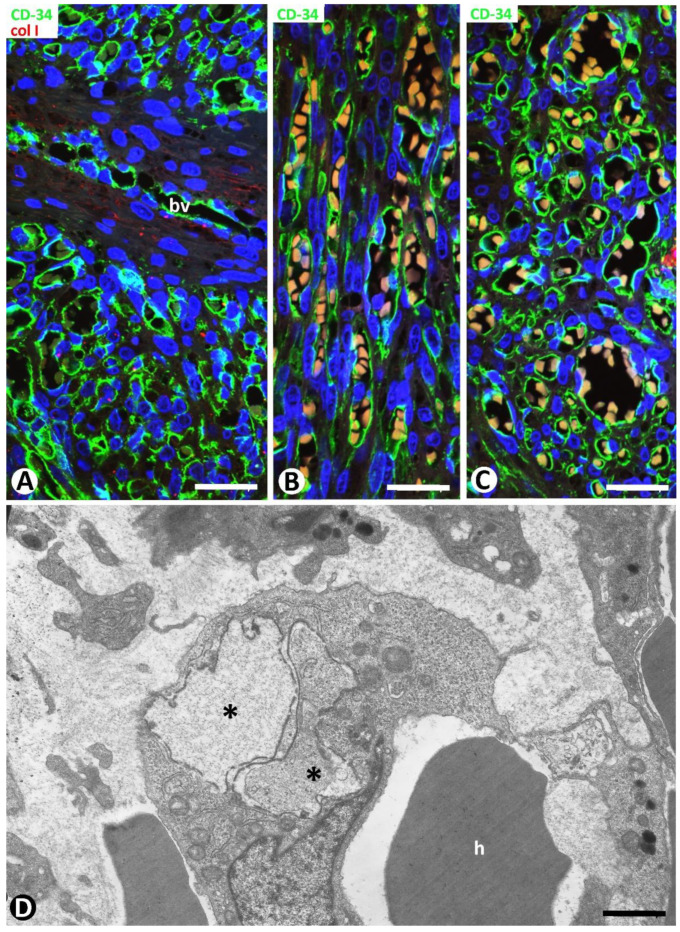
(**A**–**C**): Numerous type 2 neovessels (CD34+ ECs, green) and scarce and weak expression of collagen I (red) are observed in the nodular stage of KS. Note in (**A**) the interposed pre-existing blood vessel (bv), and in (**B**,**C**) one or several red blood cells in the lumen of different size neovessels, in longitudinal and transversal sections. (**D**): Ultrastructural image of a neovessel with an intraluminal red blood cell (h) and an EC showing intracytoplasmic degraded red blood cells (asterisks). (**A**): Double immunofluorescence for CD34 (green) and collagen I (red). (**B**,**C**). Immunofluorescence for CD34. DAPI counterstain (**D**): Ultrathin section. Uranyl acetate and lead citrate. Bar: (**A**–**C**): 25 µm; (**D**): 1.5 µm.

Numerous neovessels, predominantly type 2, interposed the pre-existing blood vessels, and intra- and extraluminal red blood cells were usual components in the nodular stage of KS (Figure 13A–C). Intracytoplasmic incorporation of degraded blood cells by the ECs was frequent (Figure 13D). Some mononuclear inflammatory cells and scarce collagen material were also seen.

### 2.5. Observations through Serial Histologic Sections

**Figure 14:**
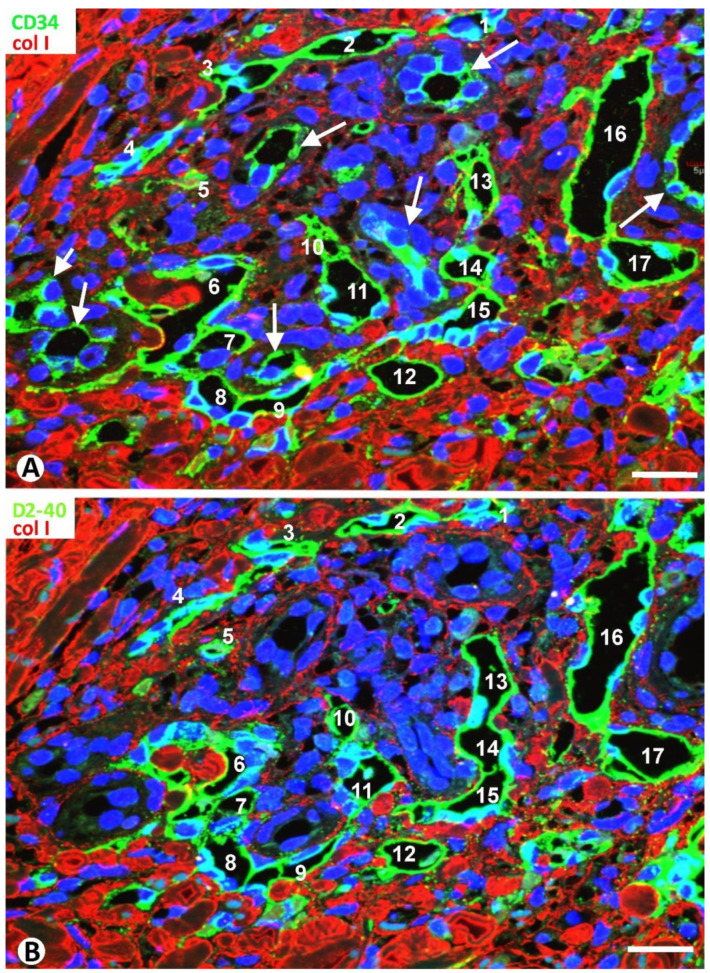
Two images were obtained from serial sections for confocal microscopy, in which the neovessels (1–17) are observed expressing CD34 in (**A**) and D2-40 in (**B**), while the pre-existing blood vessels (arrows) are positive for CD34 (**A**) and negative for D2-40 (**B**). Note how the arrangement of neovessels around pre-existing blood vessels is similar to that of CD34+SCs/TCs, as well as the evident neovessel lumens alternating with slit-lumens or small spaces. A discontinuity in the lining and an area of the initial formation of a fold centered by collagen are observed in neovessel 6 in (**A**), while a complete lining and an area of the well-formed fold are present in the same vessel in (**B**). The neovessels 13,14,15 that appear isolated in (**A**) form a continuous lumen in (**B**) as they converge. (**A**): Double immunofluorescence for CD34 (green) and collagen I (red). (**B**): Double immunofluorescence for D2-40 (green) and collagen I (red). DAPI counterstain. Bar: 40 µm.

**Figure 15:**
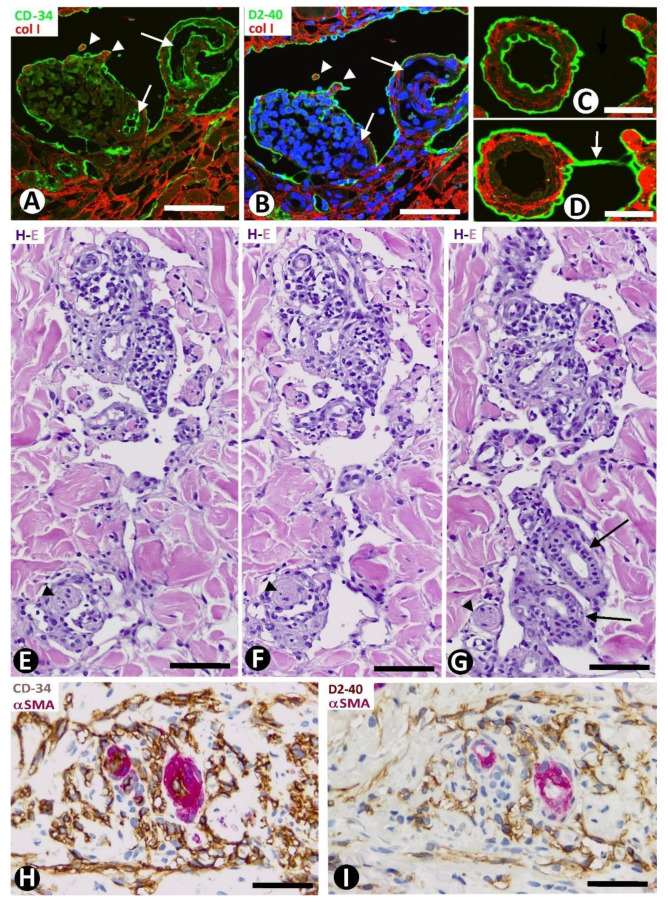
(**A**,**B**): Two large folds containing pre-existing blood vessels, showing CD34 positivity in (**A**) and negativity for D2-40 in (**B**) (arrows), project into a surrounding wide type 1 neovessel (vessel within vessel: promontory sign). Note the presence of infiltrate inflammation around pre-existing blood vessels, increasing the size of the fold, and two small pillars centered by collagen I forming on the fold surface (arrowheads). (**C**,**D**): The appearance and disappearance of a pillar observed in two adjacent sections. (**E**–**G**): A large pathway of type 1 neovessels around blood vessels, glands and a nerve. Note the appearance, disappearance and modifications of some structures in the serial sections. (**H**,**I**): Elongated type 2 neovessels are seen around pre-existing blood vessels in two of the serial sections, showing small or virtual lumens and expression of markers for ECs of both blood and lymphatic vessels. Double immunofluorescence for CD34 (green) and collagen I (red) in (**A**,**C**) and for D2-40 (green) and collagen I (red) in (**B**,**D**). Hematoxylin–eosin staining in (**E**–**G**). Double immunochemistry for CD34 (brown) and SMA (red) in (**H**) and for D2-40 (brown) and SMA (red) in (**I**). Bar: (**A**,**B**): 50 µm; (**C**,**D**): 35 µm: (**E**–**I**): 70 µm.

In the serial histologic sections with different immunomarkers in each, the following findings were specified in type 1 neovessels (Figure 14, compare A and B, Table 1), including their location around blood vessels, similar to that of the delimiting CD34+SCs/TCs; their constant expression of markers for both blood and lymphatic vessels, different from that of pre-existing blood vessels; the presence of evident vascular spaces alternating with slit-like vascular lumens, as well as with CD34+SCs/TCs, forming small lumens or not; the existence of discontinuities in the neovessel lining; the formation of intravascular folds centered by collagen; and the convergence of neovessels that appear isolated in other histologic sections. Vessels within vessels were observed when wide neovessels converged around pre-existing blood vessels (a promontory sign) (Figure 15A,B). Likewise, in adjacent sections, the appearance and disappearance of pillars (hallmarks of intussusceptive angiogenesis) were demonstrated (Figure 15C,D). Serial sections, more easily obtainable using conventional techniques with hematoxylin–eosin staining, showed longer neovessel pathways arranged around the pre-existing vessels, nerves and glands, presenting regions that appeared isolated and/or modified in other sections (Figure 15E–G). The predominant presence of small or virtual lumens in the elongated type 2 neovessels around pre-existing blood vessels, expressing markers for ECs of both blood and lymphatic vessels, was also demonstrated in the serial sections (Figure 15H,I, Table 1).

## 3. Discussion

In KS of the skin, we contribute (a) the participation of the delimiting CD34+SCs/TCs in the external layer of pre-existing blood vessels and around skin appendages in the formation of neovessels, behaving as resident mesenchymal/stromal cells capable of transdifferentiation into neovessel ECs and (b) the formation of two main types of initial neovessels in the early stages of KS, leading to progressive sarcomatous lesions.

The distinction between pre-existing blood vessels and initial neovessels was important to our observations on the evolution of delimiting CD34+SCs/TCs during their transformation into neovessel ECs in the early KS lesions. This distinction was based on the expression in their ECs of only markers for blood vessels and the presence of prominent αSMA+ mural cells (pericytes or vascular smooth muscle cells) in pre-existing blood vessels versus the expression of markers for ECs of both blood and lymphatic vessels and the absence of mural cells in the initial neovessels.

**Figure 16:**
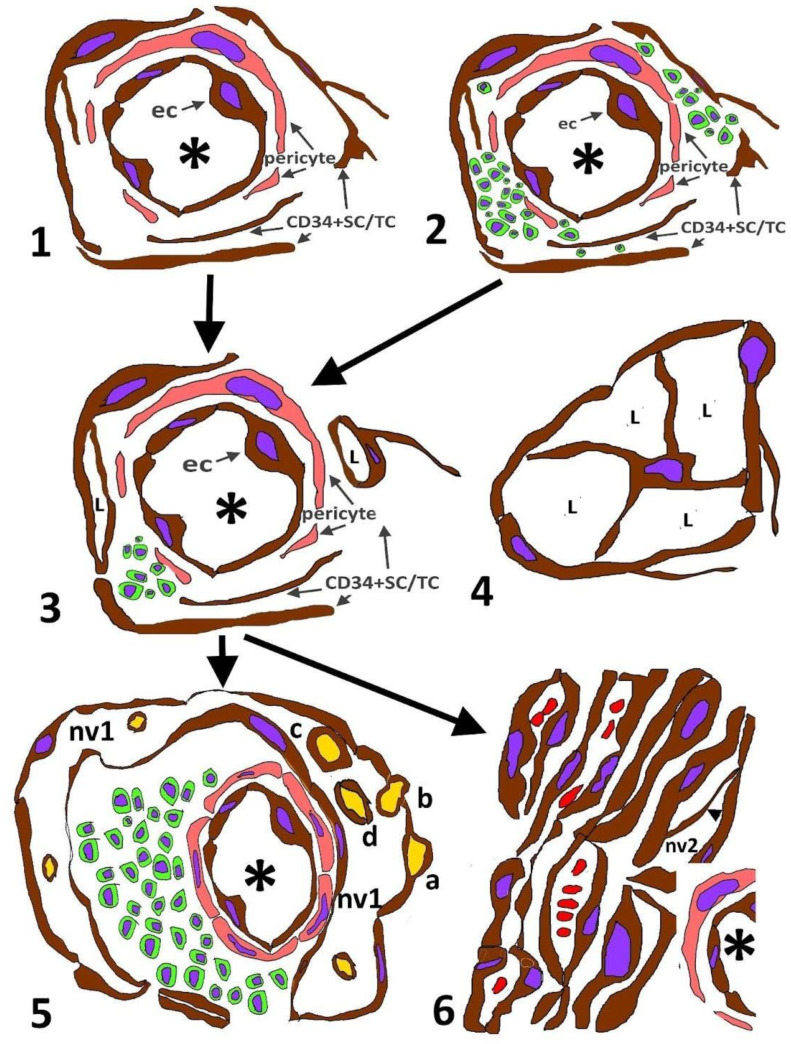
Events of CD34+SCs/TCs during the formation and evolution of neovessels in KS. (**1**): CD34+SCs/TCs in the external layer of unaffected pre-existing blood vessels (asterisk). (**2**): Perivascular inflammatory infiltrate (cells in green) encompassed by CD34+SCs/TCs. (**3**): Formation of small lumens (L) by folded and semi-detached CD34+SCs/TCs. (**4**): Detail of a neovessel with an intraluminal stellate CD34+SC/TC, originating new lumens (L). Note that pericytes are absent in the neovessel. Transitional events in the expression of markers and of ultrastructural characteristics between CD34+SCs/TCs and neighboring neovessel ECs occur in this phase, as well as the formation of two types of initial neovessels. (**5**): Evolved neovessels type 1 (nv1). Observe a thick fold, including a pre-existing vessel, within a neovessel (promontory sign). Sequential formation of pillars (a,b,c,d) from EC processes surrounding collagen (yellow) until its transport to the neovessel lumen. (**6**): Evolved neovessels type 2 (nv2) neighboring to a pre-existing neovessel (asterisk). An EC bridge (arrowhead) is observed in a neovessel.

The main events of CD34+SC/TC participation in the formation of neovessels and the evolution of the latter in the KS stages can be summarized as follows (Figure 16): (1) In the very early stages of KS, CD34+SCs/TCs retain their characteristics in the external layer of pre-existing blood vessels and around skin appendages, with and without perivascular inflammatory infiltrates, which, when present, they encompass. (2) Small lumens are formed between semi-detached and folded CD34+SCs/TCs. (3) Predominantly around pre-existing blood vessels, the lumens increase in number, converge, and form evident neovessels, whose lining ECs express markers for both blood and lymphatic vessels (the formation of neovessels around pre-existing blood vessels). In this phase, transitional findings between some neovessel neighboring CD34+SCs/TCs and neovessel ECs are seen, including (a) a presence in the lumen of neovessels of stellate CD34+SCs/TCs contacting the neovessel wall, (b) neighboring CD34+SCs/TCs to neovessels showing markers for neovessel ECs, (c) a sharing of some ultrastructural characteristics of CD34+SCs/TCs by neovessel ECs (e.g., processes toward the interstitium, alternating thick and thin cytoplasmic regions and discontinuities between some of the cells lining the neovessels), and (d) the replacement of CD34+SCs/TCs around pre-existing blood vessels by lining neovessel ECs. (4) Two main types of initial neovessels are formed: types 1 and 2, respectively, showing (a) ECs with flattened or spindle-shaped morphology, (b) anastomosing irregular pathways, or an elongated and fascicular appearance, (c) lumens of varying size, generally empty or very small, with some red blood cells, and (d) intraluminal folds and mature pillars (papillae), or only intraluminal endothelial bridges (nascent pillars). (5) Type 1 neovessels reach their greatest expression in the lymphangiectatic and lymphangiomatous variants of KS, while type 2 does so in KS lesions with a sarcomatous appearance. Next, we consider these events.

The characteristics and distribution of the CD34+SCs/TCs around apparently unaffected skin vessels and appendages in early KS lesions agree with those previously described in several conditions [22,23,24,25,26,27,28,29,30,31], creating microenvironments within the tissues (a contribution to maintenance and modulation of local homeostasis) [21,31,38,39,40,41,42,43,44,45,46,47,48,49,50,51,52]. Likewise, the ability of CD34+SCs/TCs to envelop perivascular inflammatory infiltrates in KS is also consistent with their role in retaining and modulating the immune cells [31,53,54,55,56,57], allowing us to explain the presence of an inflammatory component around some intussusceptive pre-existing blood vessels within neovessels (vessels into vessels: a promontory sign in KS).

For the transitional events between the CD34+SCs/TCs and ECs, the following issues should be considered: (a) The intraluminal persistence in the neovessels of stellate CD34+SCs/TCs keeping attachment points of their processes with the lining neovessel cells is compatible with a mechanism of vessel lumen formation by separation of adjacent CD34+SCs/TCs. In addition, the intraluminal CD34+SCs/TCs and their multiple or bipolar processes can also participate in the creation of new intraluminal spaces in the neovessels, increasing neovessel numbers by vessel splitting (intussusceptive angiogenesis). Therefore, the partial separation of adjacent and contacting CD34+SCs/TCs is an additional mechanism to those previously proposed in intussusceptive angiogenesis [58,59,60,61,62,63,64,65]. (b) The coincident expression of markers for the blood and lymphatic vessels in neovessel ECs, and in some neighboring and interspersed CD34+SCs/TCs during neovessel formation, coincides with the ability of the latter to gain new markers [22,34,35,36,37] and in this case for ECs, of blood and lymphatic vessels (c). While the existence of some neovessel ECs retaining the CD34+SC/TC characteristics supports the transition from one cell type to another, it is also an exponent of the defining ultrastructural signs of the telocytes [20,21]. (d) The total replacement of CD34+SCs/TCs around pre-existing blood vessels with neovessel ECs could be due to CD34+SC/TCs’ participation as progenitor cells or simply to their loss [31]. The absence of the degenerative signs in CD34+SCs/TCs during neovessel formation is incompatible with the latter possibility. This replacement is, therefore, another indication for their participation in neovessel formation.

The set of events outlined above supports previous studies by other authors on the participation of mesenchymal/stromal cells in the origin of KS cells [14,15,16,17] and establishes the precise location and characteristics of the resident cells (delimiting CD34+SCs/TCs) with this mesenchymal capacity. However, the fact that CD34+SCs/TCs are a source of neovessel ECs in KS does not exclude the participation of ECs in this origin, mainly of lymphatic vessels, which is also supported by the immunoprofile of neovessel ECs [6,7]. The origin of different progenitor cells concurs with the evidence for multiclonality in KS [3,4] and would lead to the connection of and interrelationship between the CD34+SCs/TCs and blood and lymphatic ECs during neovessel development. The CD34+SC/TC and EC connections with pre-existing blood vessels could facilitate the extravasation of red blood cells with hemosiderosis (abnormal neovessels connecting to blood vessels). However, one or several extravasated red blood cells could be surrounded by the CD34+SCs/TCs as described for the neoplastic cells of lobular carcinoma of the breast, in which the surrounding CD34+SCs/TCs originate cancer-associated fibroblasts [66]. Likewise, the edema retained by the CD34+SCs/TCs around pre-existing blood vessels could be partially removed by connecting lymphatic vessels (KS-associated edema). All these issues require further studies.

The formation of two main types of initial neovessels and the predominance of one in lesions with lymphangiomatous characteristics and of the other with a sarcomatous aspect during KS evolution suggests it is related to lesion variants.

Papillae in vessel lumens have been described in several tumors and pseudotumors of blood and lymphatic vessels [67,68,69,70,71,72], including KS lesions [18,19]. In previous works, we have demonstrated the similarity between these papillae and the folds and pillars that are the hallmarks of intussusceptive angiogenesis and lymphangiogenesis [63,64,66,73,74], highlighting their role in the growth of vascular networks and the morphogenesis of vessel tumors/pseudotumors [66,70,74]. In this case, CD34+SCs/TCs and their processes surrounding collagen fibers separate from the interstitium, travel into the vessel lumen and form the cover and core of folds and pillars. This mechanism resembles that described for ECs and collagen as inverse sprouting [65]. When the intraluminal fold cores contain blood vessels, with or without perivascular inflammatory infiltrates, they appear as vessels within vessels, which can explain the promontory sign described in some types of KS lesions [18,19,75,76].

This work has a histogenic and potential clinical and therapeutic interest. Indeed, it contributes to a better knowledge of the type and location of resident stromal precursor cells with a vasoformative capacity in KS, which could make it easier to obtain precursor cells for in vitro studies and assess their modulation during tumor development. It could also be the basis for future studies that confirm the possible evolution of the early lesions toward the different variants of KS and, therefore, their prognostic projection. These studies require larger series of cutaneous KS cases to monitor histological lesions over time, patient evolution and the application of new techniques, such as histomorphometric analysis, which other authors have used on telocytes [77,78].

## 4. Material and Methods

### 4.1. Human Tissue Samples

The archives of Histology and Anatomical Pathology of the Departments of Basic Medical Sciences of La Laguna University, University Hospital, and Eurofins^®^ Megalab–Hospiten Hospitals of the Canary Islands were searched for cases of KS. Specimens (paraffin blocks) were obtained from 49 cases. The patients were Caucasian: 28 males and 21 females, aged 17–88 years. All specimens were studied by conventional histologic techniques using hematoxylin–eosin staining to select blocks and more demonstrative areas in the histological sections. Of them, 30 cases were used for the immunochemistry procedures and immunofluorescence in confocal microscopy, and four cases were used for electron microscopy. A combination of different immunomarkers in the immunohistochemistry and immunofluorescence procedures and ultrastructural observations were used to establish the general characteristics of the CD34+SCs/TCs and to assess their participation in neovessel formation. Serial histological sections with different immunomarkers in each, including the observations in confocal microscopy, were undertaken to confirm the previously suggested findings. Ethical approval for this study was obtained from the Ethics Committee of La Laguna University (Comité de Ética de la Investigación y de Bienestar Animal, CEIBA 2022- 3192), including the dissociation of the samples from any information that could identify the patient. The authors, therefore, had no access to identifiable patient information.

### 4.2. Light Microscopy

The specimens were fixed in a buffered neutral 4% formaldehyde solution, embedded in paraffin, and cut into 3 µm-thick sections. The sections were deparaffinized, hydrated and stained with haematoxylin and eosin (H&E).

### 4.3. Immunohistochemistry and Immunofluorescence

Immunohistochemistry (automated and manual procedures) and immunofluorescence were carried out as described elsewhere [37,66]. For the automated immunohistochemistry procedure, sections were incubated with the following primary antibodies: anti-CD34 (Bond™ PA0212; Leica Biosystems, Newcastle, UK), anti-αSMA (Bond™ PA0943; Leica Biosystems, Newcastle, UK), Ki-67 (Bond™ PA0118; Leica Biosystems, Newcastle, UK), anti-CD31 (Bond™ PA0250; Leica Biosystems, Newcastle, UK), anti-CD45 (Bond™ PA0212; Leica Biosystems, Newcastle, UK), podoplanin monoclonal mouse anti-human, clone D2-40 (dilution 1:100), (Dako, Glostrup, Denmark) catalog No. M3619; anti-HHV-8 (13B10, mouse monoclonal, code nº. 760-4260, Roche), anti-ERG (EPR3864, rabbit monoclonal, code nº. 790-4576, Roche). For the double immunostaining carried out by the automated procedure ones, the following combination of antibodies was used: CD34/αSMA, D2-40/Ki-67 and D2-40/CD45. For the non-automated procedure, rabbit polyclonal anti-CD34 (1/100 dilution, code nº. A13929, AB clonal) and mouse monoclonal anti-αSMA (1/100 dilution, code nº. ABK1-A8914, Abyntek Biopharma) were used. For immunofluorescence, the sections were incubated with the following primary antibodies: rabbit polyclonal; anti-CD34 (1/100 dilution, code nº. A13929, AB clonal), anti-collagen type I (1/100 dilution, code nº. AB749P, Millipore) and anti-LYVE (1/200 dilution, code nº. 14917, Abcam), and for the mouse monoclonal antibodies: anti-αSMA (1/100 dilution, code nº. ABK1-A8914, Abyntek Biopharma), anti-podoplanin D2-40 (ready to use, code nº. IR072, clone D2-40, Dako) and anti-CD34 (ready to use, class II, clone QBEnd 10, code nº. IR632). For the double immunofluorescence labeling, sections were incubated, combining each polyclonal antibody with the monoclonal ones. As secondary antibodies, we have used those based on the primary ones and the desired color combination, being the following: biotinylated goat anti-mouse IgG (1:300, Calbiochem, cat. No. 401213, Calbiochem), Alexa Fluor 488 goat anti-rabbit IgG (H + L) (1:300, cat. No. A11001, Invitrogen), biotinylated goat anti-rabbit IgG (H + L) (1:500, Code: 65-6140, Invitrogen, San Diego, CA, USA), Alexa Fluor 488 goat anti-mouse IgG (H + L) antibody (1:500, Code: A28175, Invitrogen) and Streptavidin Cy3 conjugate (1:500, Code: SA1010, Invitrogen). Nuclei were stained with DAPI (4′-6′ Diamidino-2-phenylindole, dihydrochloride) (Invitrogen, D1306, 1:5000). Fluorescence immunosignals were obtained using a Fluoview 1000 laser scanning confocal imaging system (Olympus Optical). The omission of incubation in the primary antibody was used as a negative control.

### 4.4. Electron Microscopy

Electron microscopy for the ultrastructural study was carried out, as described previously [37]. The specimens were initially fixed in 2% glutaraldehyde in 100 mM sodium cacodylate buffer, pH 7.4, for 6 h at 4 °C, and subsequently, washed in the same buffer, post-fixed for 2 h in 1% osmium tetroxide, dehydrated through increasing concentrations of ethanol (40–100%) and embedded in Spurr’s resin. Ultrathin sections were collected on nickel grids, double stained with uranyl acetate and lead citrate and examined with the JEOL^®^ 100B and JEM 1011 Akishima, Tokyo, Japan, electron microscopes.

### 4.5. Quantitative Analysis

Quantitative analysis of the neovessels was performed in cases of KS in which immunochemistry (D2-40) was used (*n*: 30). The percentages in neovessels type 1 and type 2 are reported as a mean of each group and +/− standard deviation, and the early and advanced stages were analyzed by the Student’s t-test, considering a statistically significant *p*-value of less than 0.05.

## 5. Conclusions

In conclusion, in KS of the skin, we demonstrate that CD34+SCs/TCs, mainly in the external layer of pre-existing blood vessels, are resident mesenchymal cells capable of transdifferentiation into ECs. We establish: (a) the transitional findings between both types of cells, including the presence of CD34+SCs/TCs expressing markers for the ECs of blood and lymphatic vessels and the replacement of CD34+SCs/TCs by the EC of neovessels; (b) the possible interrelationships of the CD34+SC/TC descendants with ECs from other sources; (c) the formation of two types of neovessels and their evolutionary phenomena, suggesting their role in the origin of the different variants of KS with lymphangiomatous or spindle-cell patterns; and (d) the subsequent neovessel growth by mechanisms of intussusception (intussusceptive angiogenesis and lymphangiogenesis), all of which are of histogenic, clinical, and therapeutic interest.

## Figures and Tables

**Table 1:** Data refer to the cases in which immunochemistry was performed (*n*: 30). Standard deviation in percentages of the groups of early and advanced stages were 12.22 and 8.02, respectively. Early stages vs. advanced stages: significant. *p* < 0.05.

Differential Findings					Common Findings
	Endothelial Cell Characteristics	Vessel Lumen	Percentages		and Relationship with Pre-Existing Blood Vessels
			Early Stages	Advanced Stages	
Type 1 neovessel	Flattened	Irregular			- Location around pre-existing blood vessels - Absence of αSMA+ pericytes and vascular smooth muscle cells - Expression of markers for ECs of blood and lymphatic vessels (e.g., CD-34 and D2-40 positivity in all cases)
	Discontinuities	Lymphangiomatous aspect in advanced stages	56.77%	13.70%	
	Processes surrounding perivascular collagen.	Frequent folds, pillars and promontory sign			
Type 2 neovessel	Fusiform (spindle cell-type)	Elongated, virtual or small	43.23%	86.93%	
	Formation of rows (Fascicular aspect)	Frequent intraluminal red blood cells in advanced stages			

## Data Availability

All data are reported in the present paper.
